# Multiple Unhealthy Behaviors Share Equivalent Profiles of Readiness for Change in Patients with Type 2 Diabetes

**DOI:** 10.3390/ijerph18073631

**Published:** 2021-03-31

**Authors:** Ana María Salinas Martínez, Ruth Isabel Gómez Campusano, Hid Felizardo Cordero Franco, Karen Abigail Chávez Barrón, Cecilia Janeth Gutiérrez Sauceda, Francisco Javier Guzmán de la Garza, Georgina Mayela Núñez Rocha

**Affiliations:** 1 School of Public Health and Nutrition, Autonomous University of Nuevo Leon, Monterrey 64460, Mexico; ruth.gomezcam@gmail.com (R.I.G.C.); mayela6591@hotmail.com (G.M.N.R.); 2 Epidemiologic and Health Services Research Unit, Mexican Institute of Social Security, Monterrey 64360, Mexico; dr_hid_cordero@hotmail.com (H.F.C.F.); arukas.karen@gmail.com (K.A.C.B.); cecyy.gtz17@hotmail.com (C.J.G.S.); fcojguzman@hotmail.com (F.J.G.d.l.G.); 3 School of Dentistry, Faculty of Health Sciences, Universidad Nacional Pedro Henríquez Ureña, Santo Domingo 1423, Dominican Republic; 4 School of Medicine, Autonomous University of Nuevo Leon, Monterrey 64460, Mexico

**Keywords:** health behavior, diabetes, stages of change, transtheoretical Model

## Abstract

Few studies have considered more than one behavior, despite the tendency towards multiple behaviors, and there are none that have focused on a Latino population. We determined the concurrence of four unhealthy behaviors related to glycemic control and identified common cognitive factors at advanced stages of readiness for change in patients with type 2 diabetes treated in primary care. A cross-sectional study was carried out during August–December 2018 in northeastern Mexico. We consecutively included patients between 20 and 70 years who were without medical contraindication, physical impediment against exercise, pregnancy and edentulism, among other selection criteria (*n* = 407). Stages of behavior were measured according to the Transtheoretical Model. Pros, cons, self-efficacy, susceptibility, and severity data were collected by interview. Statistical analysis consisted of descriptive statistics and multiple logistic regression. A total of 36.7% exhibited more than one unhealthy behavior in precontemplation or contemplation (no interest or some interest in changing consumption of refined sugars and saturated fats, exercise, or oral hygiene behavior). Cons (*p* < 0.05) and self-efficacy (*p* < 0.001) were common to all four unhealthy behaviors, independent of potential confounders. Studies like ours facilitate the recognition of individuals with multiple unhealthy behaviors who share equivalent profiles of readiness for change before implementing public health programs.

## 1. Introduction

Diabetes has reached epidemic proportions worldwide, and suboptimal glucose levels have caused 2.2 million additional deaths due to increased cardiovascular risk and other complications [1]. In Mexico, the prevalence of diabetes increased from 7.2% in 2006 to 9.4% in 2016 [2]. Furthermore, during the COVID-19 pandemic, diabetes has been identified as a condition associated with greater virus-related severity and mortality, mainly in patients with uncontrolled glucose levels [3,4]. Several models have been proposed to explain success in behavioral change. The Transtheoretical Model (TTM) states that not everyone is equally ready to initiate a positive behavior or abandon a negative one. This model identifies at least four stages, ranging from a lack of interest in changing (precontemplation) to interest in changing (contemplation), recent change (action), and lasting change (maintenance) [5]. Precontemplation and contemplation are considered early stages, while action and maintenance are considered late stages. Traditionally, health providers have promoted healthy habits without regard for the stages of behavior, a practice that can function in individuals who are in the stage of contemplation, but not in those who are in the stage of precontemplation. The latter has a different motivational profile, and there is still no preparation to accept or implement the desired behavior. To understand progress in stages of readiness for behavioral change, the TTM recognizes the participation of self-efficacy and favorable (pros) and unfavorable beliefs (cons), while the Health Belief Model recognizes the perception of disease susceptibility and severity [6]. Thus, an advance in stage would be expected with an increase in self-efficacy, pros, susceptibility and severity, and a decrease in cons. The segmentation of the target population according to the stage of readiness and cognitive profile favorable to change is very useful because it offers the advantage of rendering actions to prevent or postpone the development of complications from diabetes more efficient.

Compliance with a set of behaviors is critical for metabolic control and to prevent or delay diabetes complications. Low consumption of refined carbohydrates and added sugars can improve glucose and lipid consequences, and a diet rich in mono and polyunsaturated fats reduces the risk of cardiovascular disease [7]. Exercise increases insulin sensitivity and can help control the levels of blood pressure and lipids, and it also favors weight loss [8], while good oral hygiene behavior prevents oral infections that can produce alterations in glycemic control [9]. The TTM has been used in patients with diabetes since 1993 to analyze behavior related to nutrition [10,11], exercise [12] and treatment adherence [13]. Despite the tendency towards multiple behaviors in adults [14], few studies considered more than one behavior at the same time, and none have focused on a Latino population [15,16,17,18]. The objective of this study was to determine the concurrence of four unhealthy behaviors (consumption of refined sugars, consumption of saturated fats, insufficient or no exercise, and insufficient or no tooth brushing), and to identify common cognitive factors at advanced stages of change in patients with type 2 diabetes treated in primary care.

## 2. Materials and Methods

This cross-sectional study was carried out during August–December 2018 in northeastern Mexico. We consecutively included patients between 20 and 70 years with no pregnancy, blindness, edentulism, renal insufficiency, amputation of lower extremities, medical contraindication, or manifestation of a physical impediment to do exercise. The participants were in waiting rooms of primary care centers of a governmental health institution from seven municipal sectors of the metropolitan area of Monterrey, the third largest of the country. The size of the sample was 406, enough for a 95% confidence level and precision of less than 5% given the observed result of participants in the stage of precontemplation or contemplation between 18% and 55%. The protocol was approved by local committees of ethics and health research (18-FaSPyN-SA-18 and R-2018-1912-038) and informed consent was provided by all of the participants.

### 2.1. Stages of Readiness for Change

The definition of stages was based on the TTM recommended guidelines for diet, exercise and tooth brushing [7,19].

#### 2.1.1. Refined Sugars and Saturated Fats Consumption

The consumption of four foods known for their high content of refined sugars (sodas, coffee or drinks with sugar, pastries, and sandwich cookies) and the consumption of five foods known for their high content of saturated fat (fried or breaded foods, whole milk, bacon, whole milk cheese or cream cheese, red meat fat and chicken skin) were identified. Two response options were available: consumption of item ≥ once a week and < once a week or never. If the response was ≥ once a week, the level of interest in reducing the frequency, portion size, or replacement with a lighter version was identified (yes = contemplation stage, no = precontemplation stage). If the response was < once a week or never, the amount of time was solicited (<6 months = action stage; ≥6 months = maintenance stage). Stages of behavioral change were measured by food and food groups high in refined sugars or saturated fats, the latter based on the mode. In the case of a bimodal distribution, the earliest stage was considered, in the absence of a mode, the median was considered.

#### 2.1.2. Exercise

The weekly frequency and duration of light to moderate (e.g., walking slowly or with pauses) or intense exercise (e.g., classes of Zumba, aerobics, spinning, yoga) were identified. There was an initial question with two response options: never practices, and practices light/moderate exercise or intense activity. If the participant did not engage in exercise, the degree of interest in starting was identified (yes = contemplation stage, no = precontemplation stage); if the response was positive to light/moderate exercise, they were asked whether the duration was <150 min or ≥150 min per week. If the duration was <150 min they were asked whether they wanted to increase it (yes = stage of preparation in contemplation, no = stage of preparation in precontemplation) and if it was ≥50 min they were asked how long they had been doing it (<6 months = action stage; ≥6 months = maintenance stage). The algorithm for intense activity was equivalent but considering 75 min of dedication.

#### 2.1.3. Tooth Brushing

The daily frequency of tooth brushing was identified. Two response options were available in relation to daily frequency of brushing: Never or <2 times a day, and ≥2 times a day. If the response was never or <2 times a day, participants were asked if they wanted to increase the frequency (yes = contemplation stage; no = precontemplation stage). If the response was ≥2, they were asked how long they had been doing it (<6 months = action stage; ≥6 months = maintenance stage).

### 2.2. Cognitive Factors

The phase of content validity consisted of identification in the literature of items on cognitive factors. A panel of public health experts then eliminated duplicate items and selected five questions per category after a thorough examination of the contents. The proposed items were then translated back to English to verify their equivalence to the content in the original language.

#### 2.2.1. Pros and Cons

Five pros (e.g., “I believe it is beneficial to remove the fat from the meat or the skin of the chicken”) and five cons (e.g., “I feel weak when I stop drinking soda or sugary drinks”) per behavior were initially proposed, but the exploratory factor analysis showed less than five items with factor loadings of ≥0.30 (construct validity). Therefore, three pros and four cons could be included. Alphas were as follows: pros and cons of consumption of refined sugars = 0.42 and 0.54, respectively; consumption of saturated fats = 0.43 and 0.51, respectively; exercise = 0.51 and 0.53, respectively; and tooth brushing = 0.49 and 0.52, respectively. Likert scale response options were used (4 = a high amount).

#### 2.2.2. Self-Efficacy

Self-confidence for changing the unhealthy behavior was identified (e.g., “I feel capable of changing from sodas with sugar to light sodas”). Likert scale response options were used (4 = very capable). The exploratory factor analysis revealed five items per behavior with factor loadings of ≥0.30. Alphas were as follows: self-efficacy for changing consumption of refined sugars = 0.76; self-efficacy for changing consumption of saturated fats = 0.73; self-efficacy for exercise = 0.90; and self-efficacy for tooth brushing = 0.90.

#### 2.2.3. Susceptibility and Severity

Participants responded to five questions related to how much they believed they could develop a complication such as kidney disease, foot circulatory failure, vision loss, gum disease and high blood pressure using a Likert scale (4 = strong belief; α = 0.81). They also responded to five questions about how severe they believed that complication was (4 = very severe; α = 0.81).

### 2.3. Other Variables

Time since diagnosis, type of treatment, latest fasting glucose reading (self-reported), history of hypertension or dyslipidemia, frequency of visits to the physician for diabetes control and to the dentist for examination in the last year, family experience with complications of diabetes, family habits of avoiding foods high in fats or sugar, exercising, or having annual visits to the dentist; age, sex, marital status, educational level, and occupation.

Three interviewers—a graduate student in Public Health Sciences and two medical graduates doing their social service—collected data by interview after receiving training in the contents of the questionnaire and interviewing techniques. At the end of the interview, the participants were weighed in kilograms using a Taylor (Oak Brook, Chicago, IL, USA) portable digital scale that was calibrated daily, and their height was measured in centimeters with a wall-mounted stadiometer. Measurements were taken without shoes and with light clothing, with feet together, and the heels, back, and hips touching the wall. Body mass index (weight/height^2^) was categorized as <18.5 = low weight, 18.5 − 24.9 = normal weight, 25.0 − 29.9 = overweight and ≥30 = obese.

### 2.4. Statistical Analysis

Statistical analysis consisted of descriptive statistics, the estimation of 95% confidence intervals and a multiple binary logistic regression analysis using the late stage of each behavior as the dependent variable and the cognitive factors as the independent variables, while controlling for the following potential confounders: time since diagnosis, type of treatment, comorbidity, glucose level, and sociodemographic and family background. A cognitive factor was considered a common factor when this was associated with a late stage of readiness for change in all four behaviors.

## 3. Results

The mean age of patients was 58.4 ± 8.5 years. Females with primary schooling, homemaker occupation, and marital status “with a partner” predominated in the participants (Table 1). The mean time since diagnosis was 11.0 ± 8.3 years; 56.9% received oral hypoglycemics, 17.2% insulin, 22.4% both, and 3.4% neither. Visits to the doctor for control of the disease were as follows: 75.6% visited monthly, 7.4% every 2 months, 11.9% every three months, and the rest visited every four months or more. A total of 26% reported a visit to the dentist in the last year, 58.4% reported hypertension, and 44.8% reported dyslipidemia. Almost 11% of patients were underweight or had normal weight, 36.2% were overweight, and 53.1% were obese. The mean self-reported fasting glucose level was 143.7 ± 50.7 mg/dL.

### 3.1. Distribution and Concurrence of Stages of Readiness for Change

The most frequent behavior of patients in precontemplation or contemplation was exercise and the least frequent was tooth brushing. A total of 24% and 28% of patients were in the early stages of reducing consumption of saturated fat or refined sugars, respectively; fried or breaded foods and sugary drinks ranked first (Table 2). The analysis showed a 2% early-stage coincidence in four behaviors, 11.8% in three behaviors and 20.9% in two behaviors. The combination of absence or insufficient exercise with consumption of saturated fats and refined sugars predominated when patients exhibited three unhealthy behaviors; the combination of absence or insufficient exercise with consumption of refined sugars predominated was most prevalent when patients exhibited two unhealthy behaviors; and absence or insufficient exercise predominated when there was one unhealthy behavior.

### 3.2. Cognitive Factors and Stage for Readiness for Change

**Figure 1:**
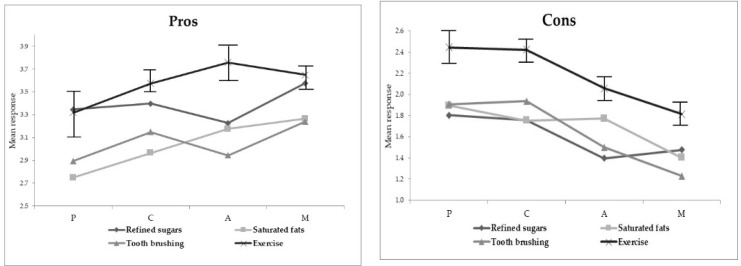

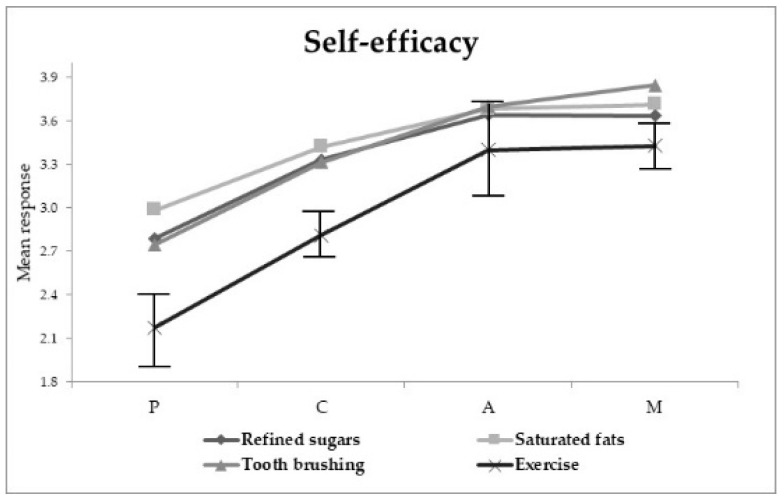
Mean and 95% confidence intervals of perception of pros, cons, and self-efficacy, patients with type 2 diabetes treated in primary care (*n* = 406) (the higher the score, the greater the perception). P = Precontemplation, C = Contemplation, A = Action, M = Maintenance.

Exercise was the behavior with the greatest perception of pros and cons, and the behavior with the lowest perception of self-efficacy in almost all stages of readiness for change (Figure 1). Detailed 95% confidence intervals are provided in a supplementary file (Table S1).

Multivariate analysis showed an association of cons and self-efficacy with late stages in all behaviors (common factors), with cons decreasing the odds of a late stage, and self-efficacy increasing them. The pros increased the likelihood of a late stage and maintained the association only in the consumption of saturated fats (specific factor) (Table 3). A total of 42% of patients believed that one day they could develop kidney disease, 55.4% foot circulatory failure, 58.1% vision loss; 52.3% gum disease; and 61.9% high blood pressure. High blood pressure was the complication most frequently perceived as very severe (46.8%), followed by vision loss (41.9%), kidney disease (37.2%), foot circulatory failure (23.9%), and gum disease (19.7%). Neither susceptibility nor severity showed an association with stages of readiness for change in any unhealthy behavior (*p* > 0.05).

## 4. Discussion

The present study was characterized by an analysis of the stages of readiness for change concerning four behaviors recommended to favor metabolic control. Two were focused on initiating a positive behavior (exercise and tooth brushing), and the other two were focused on abandoning a negative behavior (consumption of saturated fats and refined sugars) in primary care patients with type 2 diabetes. Exercise was the behavior with the highest number of participants in precontemplation or contemplation (55%). This was followed by the readiness to reduce the frequency, the portion size, or changing from products with saturated fats and refined sugars to lighter products, with 2–3 out of every 10 individuals in the precontemplation or contemplation stage. Both results were not surprising: Less than 40% of the Mexican population exercise in their free time, and this has remained stable over the last five years [20]. In Jordan, more patients were also identified as being in an early stage for exercise (78%) than refined sugars and saturated fat (37.8% and 26.9%, respectively) [17]. In the United States and Norway, the result was better for diet (79%) than for exercise (57–58%) [15,16]. Differences between countries could be due to the methodology used to define the health behavior stages or to cultural differences. For example, in Mexico, typical foods include churros, enchiladas and sopes (fried foods), and more than 80% of the population regularly drink all kinds of sugary drinks in Northern Mexico [21]. A diabetes patient should follow a diet low in saturated fats and high in fiber and complex sugars [7], for which it is critical to insist on the modification of undesirable eating habits. The behavior with the least number of patients in precontemplation or contemplation was tooth brushing: More than 80% of patients were at a later stage, although only 26.1% said they had visited the dentist for a checkup in the previous year. Daily oral hygiene and the biannual visit to the dentist are necessary habits, not only to prevent and treat oral diseases but to help control glucose [19].

The inclusion of multiple behaviors in this study made it possible to identify the combined presence of unhealthy lifestyles. The results show that 35% exhibited two, three, or four unhealthy behaviors at the precontemplation and contemplation stage, and the most frequent combination was insufficient exercise with the consumption of refined sugars or saturated fats. These data were collected before the COVID-19 pandemic, and they already indicated the need to improve healthy habits. They have always represented a challenge for primary care services, which have become even greater during the COVID-19 pandemic because of virus transmission control measures that have negatively affected eating patterns (e.g., consumption of more snacks and prepared food during home confinement) and exercise routines due to outdoor activity restrictions [22,23]. Nevertheless, multiple health behaviors, such as physical activity, fruit and vegetable intake, red meat consumption, multivitamin use and smoking cessation, can be improved simultaneously through interventions delivered in primary care and at a modest cost [24].

Patients with diabetes need to overcome motivational and attitudinal barriers to progress to higher stages of readiness for change. In this study, cons and self-efficacy were common factors of stage advance in all behaviors, and the pros were specific to the reduction of saturated fats. Self-efficacy has been shown to play an important role as a predictor of higher stages of healthy behaviors [12,25,26,27], and pros and cons have been associated with a reduction of fat in the diet [25], but the results for exercise are mixed [26,28,29]. Notably, susceptibility or severity of complications did not make a difference in stage of readiness, contrary to what the Health Belief Model proposed [6]. Other studies have also failed to show an effect of susceptibility or severity of complications on behavior change [29,30]. Perhaps this is due to misinformation or negation. It would be interesting to examine the precise reasons for this lack of association in the future.

### Limitations of the Study

This study only included patients receiving primary care in the largest governmental organization providing health services, pensions, and social security in Mexico, so caution is needed for generalizing the results to the private sector. We only included participants from primary care units located in the metropolitan area of Monterrey, a highly developed urban area. Future research should consider participants from rural environments. Nevertheless, the strength of the representation of all municipalities of the metropolitan area is worth noting. Since individuals with complications or current pregnancy were excluded, it is desirable to analyze the readiness for change in patients with these characteristics.

## 5. Conclusions

This study provides evidence on the readiness for change profiles shared by individuals with multiple unhealthy behaviors. The behavior identified as having the greatest need for change was exercise. The greatest proportion of patients were in the precontemplation or contemplation stage for this change. This was followed by the consumption of refined sugars, saturated fats, and tooth brushing. More than a third of the participants exhibited two or more unhealthy behaviors with change states in the undesirable early stages. This information is of practical use for health promotion specialists and health decision-makers for segmenting the target population according to willingness to change. The need for persuasion is greater for individuals who are unprepared to initiate a positive behavior (or abandon a negative one), compared to those who have already adopted the desired behavior. The latter will only require the encouragement to maintain the change. We also identified that cons and self-efficacy were factors associated with readiness for change in multiple unhealthy behaviors. This offers the advantage of implementing health promotion activities more efficiently because they will be focused on shared enabler factors. Public health programs are essential for benefiting compliance with behaviors that favor glycemic control in patients with diabetes.

## Figures and Tables

**Table 1:** Sociodemographic and family profile in patients with type 2 diabetes treated in primary care (*n* = 406).

Characteristic	Frequency
Sex, female	65%
Highest educational level	
None	3.9%
Primary	35.5%
Secondary	30.3%
Preparatory or Technical	21.2%
College or Postgraduate	9.1%
Occupation	
Housewife	49.3%
Employee/self-employed	30.6%
Retired	19.2%
Unemployed	1.0%
Marital status, with partner	76.6%
Family background	
≥1 family member has complications of diabetes	64.8%
≥1 family member tries to avoid foods high in fats or sugars	71.9%
≥1 family member tries to exercise	64.8%
≥1 family member visits the dentist annually for revision	69.5%

**Table 2:** Stages of readiness for change in four unhealthy behaviors, patients with type 2 diabetes treated in primary care (*n* = 406).

	Stage of Readiness for Change					
	P	C	P + C (Early)	A	M	A + M (Late)
Food high in saturated fat	Consumption ≥ once a week			Consumption never or <once a week		
	Interest in reducing frequency, portion size or change to light product			Time with this frequency		
	No	Yes		<6 months	≥6 months	
Fried or breaded food	11.9%	25.4%	37.3%	7.7%	55.1%	62.8%
Whole milk	10.3%	23.6%	33.9%	4.7%	61.3%	66.0%
Bacon or menudo (tripe)	9.9%	15.1%	25.0%	11.4%	63.7%	75.1%
Whole milk cheese or cream cheese	9.9%	17.0%	26.9%	10.1%	63.1%	73.2%
Red meat fat or chicken skin	5.7%	12.6%	18.3%	10.1%	71.7%	81.8%
Global, saturated fat	6.9%	16.7%	23.6%	8.4%	68.0%	76.4%
**Food high in refined sugars**						
Sugary sodas	8.6%	30.0%	38.6%	7.9%	54.0%	61.9%
Pastries	8.9%	25.0%	33.9%	7.9%	58.0%	65.9%
Sandwich cookies	5.2%	13.0%	18.2%	8.9%	73.0%	81.9%
Coffee or sugary fruit drinks	4.7%	14.0%	18.7%	3.5%	78.0%	81.5%
Global refined sugars	5.7%	22.4%	28.1%	5.4%	66.5%	71.9%
Tooth brushing	Never or < twice a day			≥Twice a day		
	Interest in increasing frequency			Time with this frequency		
	No	Yes		<6 months	≥6 months	
	4.7%	13.3%	18.0%	1.5%	80.5%	82.0%
Exercise	Never, moderate <150′ or intense <75′ a week			Moderate ≥150′ or intense ≥ 75′ a week		
	Interest in starting/ increasing the frequency			Time with this frequency		
	No	Yes		<6 months	≥6 months	
	24.2%	30.6%	54.8%	7.9%	37.3%	45.2%

P = Precontemplation, C = Contemplation, A = Action, M = Maintenance.

**Table 3:** Logistic regression analysis of cognitive factors and late stage of the behavior, patients with type 2 diabetes treated in primary care (*n* = 406).

	Action or Maintenance			
	Consumption of Refined Sugars	Consumption of Saturated Fats	Exercise	Tooth Brushing
	Crude odds ratio (95% confidence interval)			
Pros	1.4 (1.1, 1.9) *	1.7 (1.3, 2.3) ***	1.6 (1.1, 2.1) ***	n.s.
Cons	0.5 (0.4, 0.7) ***	0.5 (0.3, 0.7) ***	0.3 (0.2, 0.4) ***	0.1 (0.1, 0.2) ***
Self-efficacy	2.3 (1.7, 3.2) ***	3.1 (2.1, 4.6) ***	2.4 (1.9, 3.0) ***	4.5 (3.0, 6.7) ***
	Adjusted odds ratio (95% confidence interval)			
Pros	n.s.	1.4 (1.1, 2.0) *^,a,c^	n.s.	n.s.
Cons	0.6 (0.4, 0.9) *^,a,b^	0.6 (0.4, 0.9) *^,a,c^	0.3 (0.2, 0.5) ***^,a,d^	0.2 (0.1, 0.3) ***^,a,e^
Self-efficacy	2.2 (1.6, 3.1) ***^,a,b^	2.8 (1.8, 4.4) **^,a,c^	2.0 (1.5, 2.5) ***^,a,d^	2.4 (1.4, 4.0) **^,a,e^

^a^ The model was adjusted by sex, age, marital status, educational level, occupation, hypertension and/or dyslipidemia, family member with complications of diabetes, time since diabetes diagnosis, type of treatment, and self-reporting of glucose level; ^b^ The model also included ≥1 Family member tries to avoid foods high in refined sugars; ^c^ The model also included ≥1 Family member tries to avoid foods high in saturated fats; ^d^ The model also included ≥1 Family member tries to exercise; ^e^ The model also included ≥1 Family member visits the dentist annually for revision and patient visited the dentist for a checkup last year. * *p* < 0.05, ** *p* < 0.01, *** *p* < 0.001. n.s. = not significant.

## Data Availability

Not Applicable.

## Supplementary Materials

The following are available online at https://www.mdpi.com/article/10.3390/ijerph18073631/s1, Table S1: Mean and 95% confidence intervals of cognitive factors by stage of behavior, patients with type 2 diabetes treated in primary care (*n* = 406).
