# Supplementary material for: Multiple Unhealthy Behaviors Share Equivalent Profiles of Readiness for Change in Patients with Type 2 Diabetes
Source: Int J Environ Res Public Health. 2021 Mar 31;18(7):3631. doi: 10.3390/ijerph18073631 (PMC8037007; doi:10.3390/ijerph18073631)
Supplement: Supplementary file 1 [file ijerph-18-03631-s001.pdf]

International Journal of
Environmental Research and Public Health

MDPI

Table S1. Mean and 95% confidence intervals of cognitive factors by stage of behavior, patients with type 2 diabetes treated in primary care (n = 406).

Behavior	Pros	Cons	Self-efficacy
Precontemplation			
Exercise	3.3 (95%CI 3.1,3.5)	2.4 (95%CI 2.3,2.6)	2.2 (95%CI 1.9,2.4)
Consumption of food high in refined sugars	3.3 (95%CI 3.0,3.7)	1.8 (95%CI 1.5,2.2)	2.8 (95%CI 2.3,3.2)
Tooth brushing	2.9 (95%CI 2.4,3.4)	1.9 (95%CI 1.6,2.2)	2.7 (95%CI 2.2,3.2)
Consumption of food high in saturated fat	2.8 (95%CI 2.4,3.1)	1.9 (95%CI 1.6,2.2)	3.0 (95%CI 2.6,3.4)
Contemplation			
Exercise	3.6 (95%CI 3.5,3.7)	2.4 (95%CI 2.3,2.5)	2.8 (95%CI 2.7,3.0)
Consumption of food high in refined sugars	3.4 (95%CI 3.3,3.5)	1.8 (95%CI 1.6,1.9)	3.3 (95%CI 3.2,3.5)
Tooth brushing	3.1 (95%CI 2.9,3.4)	1.9 (95%CI 1.7,2.1)	3.3 (95%CI 3.1,3.5)
Consumption of food high in saturated fat	3.0 (95%CI 2.8,3.2)	1.8 (95%CI 1.6,1.9)	3.4 (95%CI 3.3,3.6)
Action			
Exercise	3.8 (95%CI 3.6,3.9)	2.1 (95%CI 1.9,1.8)	3.4 (95%CI 3.1,3.7)
Consumption of food high in refined sugars	3.2 (95%CI 2.9,3.6)	1.4 (95%CI 1.2,1.6)	3.6 (95%CI 3.4,3.9)
Tooth brushing	2.9 (95%CI 1.8,4.1)	1.5 (95%CI 0.9,2.1)	3.7 (95%CI 3.3,4.1)
Consumption of food high in saturated fat	3.2 (95%CI 2.9,3.4)	1.8 (95%CI 1.5,2)	3.7 (95%CI 3.5,3.8)
Maintenance			
Exercise	3.7 (95%CI 3.6,3.7)	1.7 (95%CI 1.9,3.7)	3.4 (95%CI 3.3,3.6)
Consumption of food high in refined sugars	3.6 (95%CI 3.5,3.7)	1.5 (95%CI 1.4,1.6)	3.6 (95%CI 3.7,3.7)
Tooth brushing	3.2 (95%CI 3.2,3.3)	1.2 (95%CI 1.3,1.2)	3.8 (95%CI 3.8,3.9)
Consumption of food high in saturated fat	3.3 (95%CI 3.2,3.4)	1.4 (95%CI 1.3,1.5)	3.7 (95%CI 3.7,3.8)
